# Hyperbaric intervention ameliorates the negative effects of long-term high-altitude exposure on cognitive control capacity

**DOI:** 10.3389/fphys.2024.1378987

**Published:** 2024-08-30

**Authors:** Hong Ren, Yun-Peng Zhu, Rui Su, Hao Li, Yong-Yue Pan

**Affiliations:** ^1^ Plateau Key Laboratory of High Altitudes Brain Science and Environmental Acclimation, Tibet University, Lhasa, China; ^2^ School of Medicine, Tibet University, Lhasa, China

**Keywords:** high-altitude exposure, hyperbaric oxygen intervention, cognitive control capacity, cognitive function, MFT-M task

## Abstract

**Introduction:**

Hypoxia due to reduced partial pressure of oxygen from high-altitude exposure affects the cognitive function of high-altitude migrants. Executive function is an important component of human cognitive function, characterized by high oxygen consumption during activity, and its level can be measured using cognitive control capacity (CCC). In addition, there is evidence for the potential value of hyperbaric oxygen (HBO) interventions in improving cognitive decline on the plateau. Therefore, the objective of this study was to investigate the effect of long-term high-altitude exposure on CCC in high-altitude newcomers and whether hyperbaric oxygen intervention has an ameliorative effect.

**Methods:**

This study measured the magnitude of participants’ CCC using a Backward Masking Majority Function Task (MFT-M). Study 1 was a controlled study of different altitude conditions, with 64 participants in the high-altitude newcomer group and 64 participants in the low-altitude resident group, each completing the MFT-M task once. Study 2 was a controlled HBO intervention study in which newcomers who had lived at a high altitude for 2 years were randomly divided into the HBO group (n = 28) and control group (n = 28). 15 times hyperbaric oxygen interventions were performed in the HBO group. Subjects in both groups completed the MFT-M task once before and once after the intervention.

**Results:**

Study 1 showed that CCC was significantly higher in the low-altitude resident group than in the high-altitude newcomer group (*p* = 0.031). Study 2 showed that the CCC in the HBO group was significantly higher after 15 hyperbaric interventions than before (*p* = 0.005), while there was no significant difference in the control group (*p* = 0.972). The HBO group had significantly higher correct task rates than the control group after the intervention (*p* = 0.001).

**Conclusion:**

This study confirms that long-term high-altitude exposure leads to impairment of CCC in high-altitude newcomers and that hyperbaric oxygen intervention is effective in improving CCC.

## 1 Introduction

The effects of long-term exposure to high-altitude, low-oxygen environments on human cognitive function mostly manifest as Mild Cognitive Impairment (MCI), with core symptoms of cognitive decompensation (de Aquino Lemos et al., 2012). This is because air pressure decreases as altitude increases under high-altitude exposure conditions. This leads to lower partial pressure of oxygen (PO_2_) throughout the human physiological oxygen transport chain. This, in turn triggers a series of physiological responses that help restore blood oxygen levels (Luks and Hackett, 2022). The process mainly involves an increase in ventilation, but also a decrease in arterial oxygen saturation, an increase in pulmonary artery pressure, and an increase in erythropoietin and hemoglobin concentrations, resulting in a dynamic hypoxic-resistant state (Weil et al., 1970; Swenson, 2013). Owing to the high demand for oxygen in the human brain, hypoxia caused by prolonged high-altitude exposure can disrupt the balance between excitatory and inhibitory processes in the brain. It affects various cognitive functions, including perception, memory, and executive functions (Ma et al., 2018; Virués-Ortega et al., 2006; Zhang et al., 2018a). It is very important to maintain better executive function for newcomers who are exploring, working, studying, and living in the highlands on a non-short-term basis. Cognitive control, also known as executive control. Good cognitive control helps people efficiently select important information from a wide variety of stimuli and prioritize it in the human brain for timely execution of that information task (McConnell and Shore, 2011).

Specifically, as the most important cognitive function dominated by the prefrontal lobe, executive function has an important influence on attention, decision-making, working memory, learning, and other cognitive functions. The brain is highly susceptible to hypoxia at high altitudes, as evidenced by reduced attention, decision-making, information processing speed, and other related executive functions (Zhang et al., 2018b). While executive function is limited by cognitive control capacity (CCC), it refers to the maximum amount of information that the human brain can simultaneously process the target information per unit time and reflects the level of cognitive control ability.

Living at a very high altitude (3,500–5,500 m) for more than 3 months is generally considered long-term high-altitude exposure. In terms of the effects on functional brain structures, one study found that transplants exposed to chronic hypoxia had reduced gray matter volumes in the bilateral anterior insula, bilateral prefrontal lobes, left precentral gyrus, left cingulate gyrus (Yan et al., 2010), and right lingual gyrus, all of which are brain structures highly associated with executive functions. This corroborates reports of reduced frontal lobe function and cortical arousal, leading to reduced alertness and other executive control abilities in high-altitude-exposed migrants (Prigatano et al., 1983; Bédard et al., 1991). It has been found that damage to the right anterior insular cortex (AIC) leads to a significant reduction in CCC (Wu et al., 2019). While executive function is limited by CCC, a hypothesis is proposed that, relative to low-altitude conditions, newcomers exposed to chronic high-altitude hypoxia will experience a decline in executive function represented by a decrease in CCC.

To quantify the CCC size one can use the Backward Masking Majority Function Task (MFT-M) developed by Fan (2014) et al. This task manipulates the entropy of the information to be controlled and the exposure time of the stimulus to quantify the speed of information processing for cognitive control to quantitatively characterize the subject’s CCC under time constraints. Normal adult CCC measured using the MFT-M task is between 3 and 4 bits per second (bps). Bps refers to the amount of information pertaining to cognitive domains that can be processed by the human brain within a given time frame. The capacity for attention, decision-making, perception, movement and sensory abilities ranges from 2 to 60 bps. The MFT-M task can be used to compare the differences in CCC between healthy adults and patients with impaired cognitive function. For example, one study found significant CCC decrements in patients with mild cognitive impairment compared to healthy adults, mainly in the areas of processing speed, flexibility, and working memory. The MFT-M task also has the potential to measure executive function under high-altitude exposure conditions. While previous high-altitude studies have focused on aspects of attentional function, response inhibition, and working memory, that is, one part of cognitive control, the MFT-M Task Measure CCC can reflect the overall ability of high-altitude transplants to perform functions, as well as pre- and post-injury changes or certain interventions.

It is feasible to intervene in plateaued cognitive decline or improve cognitive function in high-altitude newcomers by improving their oxygen environment. For example, hyperbaric oxygen (HBO) plays an important role in the prevention and treatment of altitude sickness and acute plateau disease and can also effectively improve cognitive functions such as learning and spatial memory in brain-injured animals and even humans (Harch et al., 2007; Palzur et al., 2008). In fact, the use of HBO to intervene in the CCC decline in the high-altitude executive function can yield better results. First, HBO therapy improves pulmonary ventilation and blood oxygen saturation levels in high-altitude newcomers by directly increasing the partial pressure of oxygen and reducing hypoxic tissue damage (Penaloza and Arias-Stella, 2007). Hyperbaric oxygen accelerates the dissolution of inert gases by replacing them with oxygen in bubbles, which are then rapidly utilized and metabolized by the brain tissue. For example, a prospective randomized trial in patients with mild traumatic brain injury found that HBO significantly improved cognitive impairment and metabolic brain injury, even in the chronic phase (Boussi-Gross et al., 2013). One study confirmed treatment with HBO prevents the loss and morphological deterioration of immature neurons, promotes the overall proliferation of progenitors (Jeremic et al., 2023).

More specifically, several fMRI studies have shown that HBO improves cognitive performance at high oxygen concentrations by improving performance on visual and spatial tasks, with increased brain activation in several areas of the brain (e.g., cingulate gyrus, thalamus, and superior parietal lobe). Activation of the right frontal gyrus, left temporal gyrus, and left cingulate gyrus increased when subjects performed a speech task under high oxygen concentration conditions (Chung et al., 2006). For example, hyperbaric oxygen therapy has been shown to be effective in activating neuroplasticity in stroke patients, improving memory deficits (Boussi-Gross et al., 2015), and improving executive function in stroke patients by modulating the spontaneous activity of neurons in the frontal middle gyrus and superior parietal lobule brain regions (Hadanny et al., 2020). As previously mentioned, long-term high-altitude exposure can damage the brain structures of immigrants. This implies that HBO also have the potential to improve executive function impairments in newcomers exposed to high-altitude hypoxia. Therefore, we propose that HBO intervention may improve CCC impairment among high-altitude newcomers.

## 2 The current study

**FIGURE 1:**
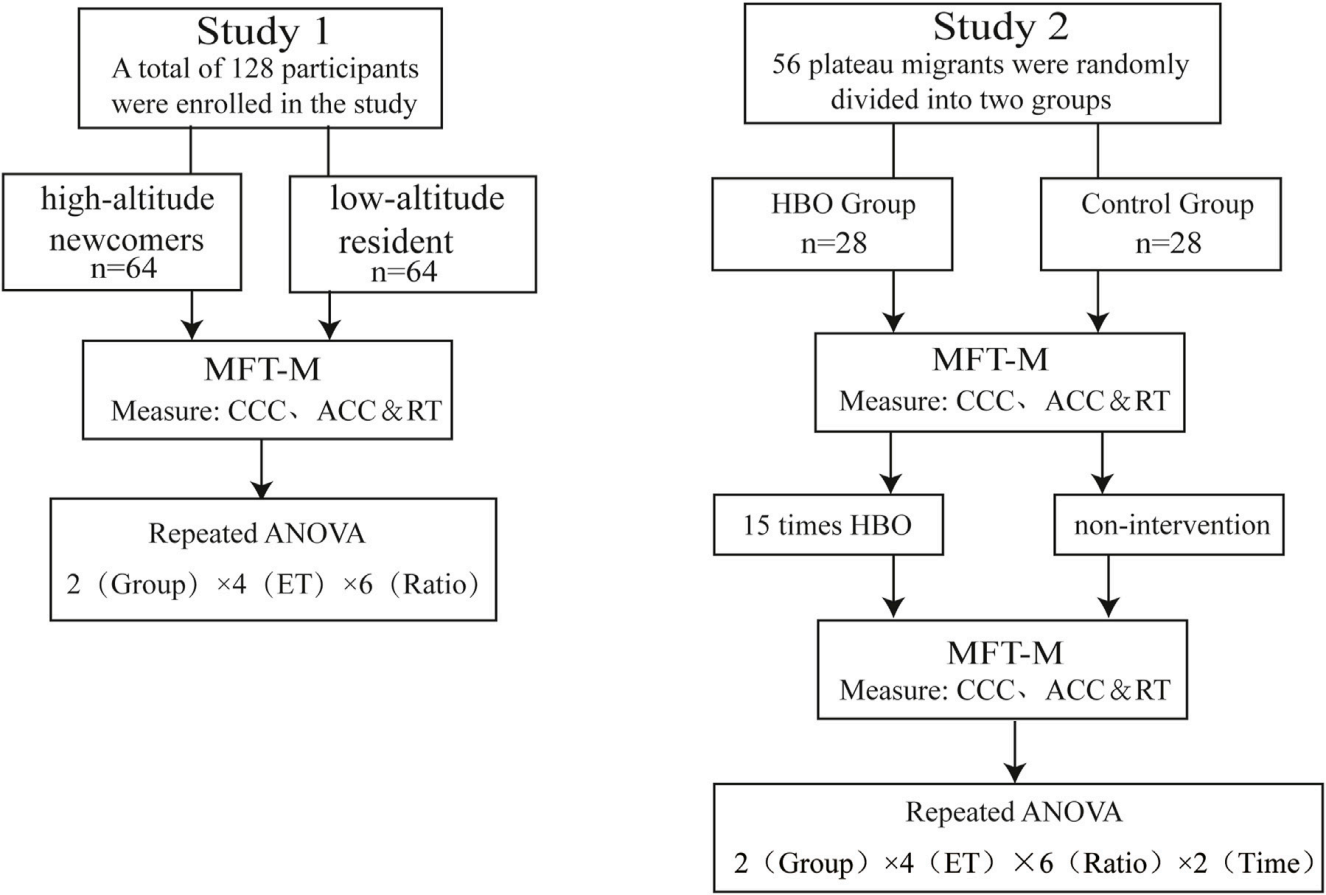
Flow chart of the experiment. Notes: abbreviations: ET, exposure time; MFT-M, Backward Masking Majority Function Task; CCC, cognitive control capacity; ACC, accuracy; RT, reaction time; HBO, hyperbaric oxygen.

To investigate the effect of long-term high-altitude exposure on the CCC of high-altitude newcomers and whether hyperbaric oxygen intervention has an ameliorative effect. We conducted two studies. Study 1 was a high-altitude and low-altitude controlled experiment to verify that high-altitude exposure negatively affects executive function in newcomers and manifests itself in differences in CCC. Study 2 was a 30-day intervention-controlled study to demonstrate the positive improvement effect of the HBO intervention on cognitive control capacity in high-altitude newcomers. In both studies, we used the MFT-M to measure the capacity of the CCC in performing the function as well as dynamic changes. Therefore, we explored whether long-term high-altitude exposure caused damage to the CCC of newcomers and assessed the CCC of participants before and after the hyperbaric oxygen intervention. (Figure 1). This experiment was approved by the Ethics Committee of Tibet University (No. XZTU2021ZRG-09).

## 3 Study 1. effect of high-altitude exposure on CCC

### 3.1 Methods

#### 3.1.1 Participants

The minimum sample size for this study was 112 according to the G*power repeated measurement analysis of variance standard principle (Faul F et al., 2009). In this study, 128 participants aged 18–20 years were divided into high-altitude newcomers and low-altitude residents, including 64 high-altitude newcomers (32 of each sex) who lived at low altitude for a long time and went to high altitude (3,650 m) for 2 years. There were 64 low-altitude residents (32 of each sex) who had never traveled to high altitudes (1,500 m and above). There were no significant differences between the two groups in terms of sex, age, or other factors (*p* > 0.05). All participants had normal or corrected visual acuity and were right-handed, signed an informed consent form before the experiment, and received some remuneration at the end of the experiment.

#### 3.1.2 Procedure

This was done while controlling for sex, age, vision, and other factors. SpO_2_ was measured for all participants prior to the MFT-M test. High-altitude newcomers and low-altitude residents completed the MFT-M task once to measure the CCC size. A three-factor mixed experimental design of 2 (groups) × 4 (ET) × 6 (ratios) was used where 1) Group: high-altitude newcomers, low-altitude residents 2). ET: arrow exposure time, including 0.25, 0.5, 1, and 2 s. The presentation time was varied by changing the presentation time of the arrows and the masking stimulus 3). Ratio: Consistency of arrows, ratio between the direction of the majority of arrows and the minority direction. It included six conditions: 1:0, 2:1, 3:1, 3:2, 4:1, and 5:0. The majority direction is where most arrows appear to point. The length and diameter of the arrow for each diamond were 0.37°, and the viewing angle from the center of the gaze point to the center of the arrow was approximately 1.5°. The between-group variable was group, and the within-group variables were ET and Ratio (Table 1). The experimental environment had good lighting, quiet and suitable temperature and humidity conditions. Behavior program computer performance is stable.

**TABLE 1:** Demographics background information for all participants.

	High-altitude newcomer (n = 64)	Low-altitude resident (n = 64)	HBO Group (n = 28)	Control Group (n = 28)
Age (years)	19.1 ± 0.58	18.6 ± 0.71	20.9 ± 1.21	21.1 ± 0.93
Sex (male/female)	32/32	32/32	14/14	15/13
Handedness (right/left/mixed)	64/0/0	64/0/0	28/0/0	28/0/0
Non-verbal IQ (Raven)	60.05 ± 3.78	59.5 ± 3.50	59.8 ± 3.20	60.2 ± 3.35
Education level (college/high school)	64/0	64/0	28/0	28/0
SpO_2_(before HBO intervention)	87.67% ± 2.45%	97.33% ± 1.25%	88.07% ± 2.57%	87.91% ± 2.32%
SpO_2_(after HBO intervention)	—	—	96.33% ± 1.29%	87.60% ± 2.56%

#### 3.1.3 Backward Masking Majority Function Task

**FIGURE 2:**
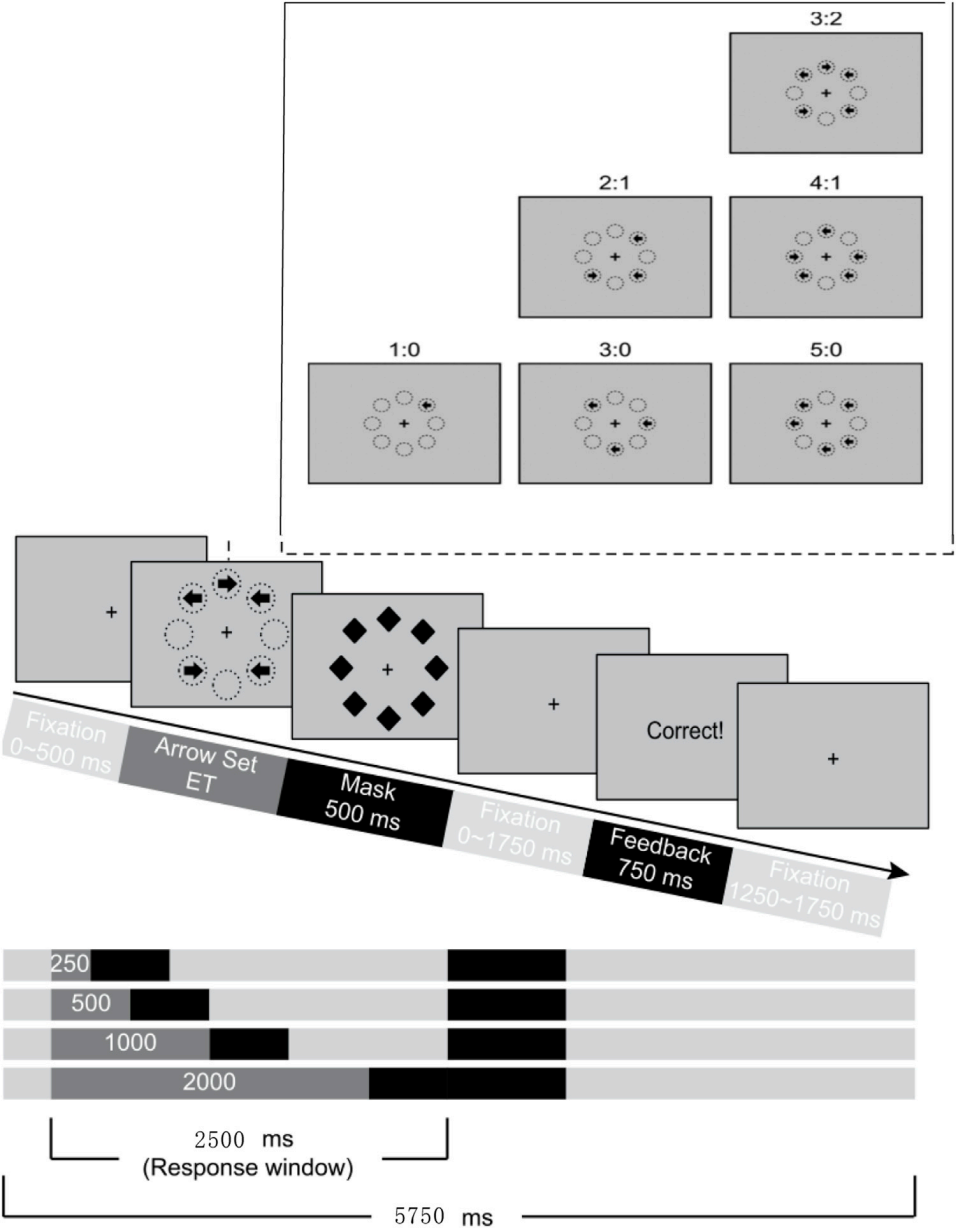
Flow chart of MFT-M.

The CCC of each participant was measured using the MFT-M task developed by Fan et al. Programmed with E-prime 2.0 and presented using a 14-inch laptop, the arrows appear randomly in any one, three or five positions on a cross-centered octagon during the task. Each arrow was randomly oriented to the left or right, and all arrows were presented simultaneously. The subject responded by pressing “F” or “J” on the keyboard after the arrows disappeared, and was asked to determine the direction of most arrows quickly and accurately. There were 12 blocks, each with 36 trials, and the four exposure times (ET) in each block were presented randomly with the same consistency of arrows. The experiment began with a randomized gaze of 0–0.5 s. After the gaze disappeared, an arrow appeared. After the arrow disappeared, a masking stimulus consisting of a diamond appears for 0.5 s. The experiment began with a randomized gaze of 0–0.5 s, and after the arrow disappeared, a masking stimulus consisting of a diamond appeared. After the masking stimulus disappears, a randomized gaze point from 0 to 1.75 s will appear, so the total duration of the arrow, masking stimulus and post-stimulus gaze point is 2.5 s. Subjects should respond within 2.5 s of the onset of the target stimulus. When the participants were unable to determine the direction of most of the arrows, they had to guess how to complete the answer. A 1–1.5 s gaze point occurred at the end of each trial, giving a total duration of 5.75 s for a full trial. At the end of each block, the subject took a break and pressed the spacebar for the next block of the experiment. A flowchart of the experimental paradigm is shown in Figure 2.

#### 3.1.4 Statistical analysis

The MFT-M behavioral data analysis was performed to calculate the mean and standard deviation of the response time and accuracy for each condition. From the perspective of information theory, information uncertainty can be quantitatively defined as the information entropy of a channel in bits, and the rate of information transmission is the average amount of information transmitted per unit time. According to the noisy channel coding theorem, if the required transmission rate exceeds its upper limit, it will result in decreased accuracy in transmitting information. In this computational model, the accuracy (ACC) declines when the amount of information exceeds the upper limit of cognitive control. Therefore, cognitive control ability can be estimated in bits per second (bps) by fitting the model to individual participants’ accuracy. Trials that did not respond to the reaction time were considered invalid. Reactions exceeding the mean plus or minus three standard deviations were considered outliers. Additionally, invalid trials and outliers were excluded from analysis. The remaining number of trials per participants was used to calculate the mean and standard deviation when responding to different conditions, and the number of correct responses per participants under each condition was used to calculate the correct rate. Data were analyzed using SPSS 20.0, and repeated- measures analysis of variance (ANOVA) was performed for reaction time (RT) and accuracy (ACC) in both studies, with Greenhouse-Geisser correction for non-sphericity results and Bonferroni correction for multiple comparisons. The analysis of CCC was calculated using the method in the published paper by Fan et al.

### 3.2 Results

**FIGURE 3:**
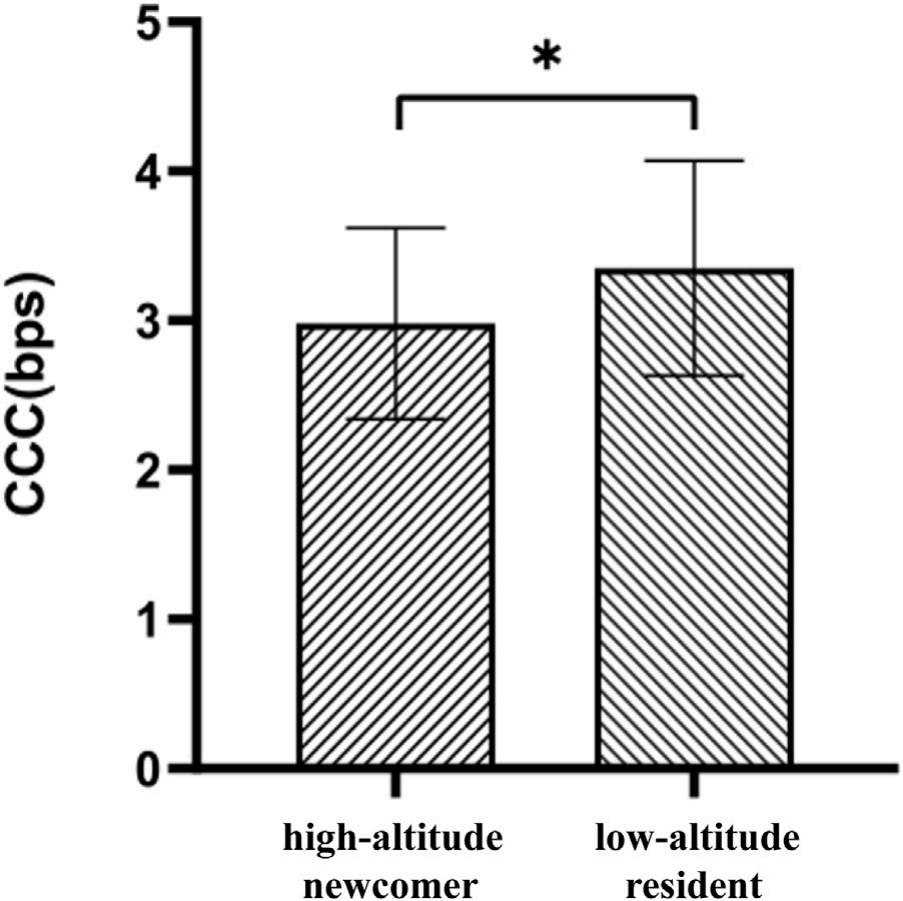
Results of cognitive control capacity for plateau settlers and plainsman. Note: *p < 0.05.

An independent samples t-test on the cognitive control capacity of high-altitude newcomers and low-altitude residents subjects showed that the mean CCC size was 2.98 (SD = 0.64) bps for high-altitude newcomers and 3.35 (SD = 0.72) bps for low-altitude residents, and it could be found that the CCC of high-altitude newcomers was significantly lower than that of low-altitude residents (t = −3.477, *p* = 0.031) (Figure 3).

A 2 (group) × 4 (ET) × 6 (ratio) repeated-measures ANOVA was performed on the correct rate (Table 2). The results showed a significant main effect of Group [*F* (1, 207) = 9.281, *p* < 0.01, *η* ^*2*^ *p* = 0.04]. The main effect of ET was significant [*F* (3, 621) = 271.622, *p* < 0.001, *η* ^*2*^ *p* = 0.57]. The main effect of Ratio was significant [*F* (5, 1035) = 1616.072, *p* < 0.001, *η* ^*2*^ *p* = 0.89].

**TABLE 2:** Accuracy and reaction time of plateau migrants and plains settlers in the MFT-M task.

	ET(s)	Ratio					
		1:0	2:1	3:0	3:2	4:1	5:0
ACC (%)							
high-altitude newcomer	0.25 0.5 1.0 2.0	96.0 (0.3) 97.6 (0.3) 98.3 (0.2) 98.2 (0.3)	68.6 (1.2) 73.8 (1.1) 83.8 (1.0) 91.0 (0.9)	97.3 (0.3) 98.4 (0.3) 99.1 (0.2) 99.0 (0.2)	60.9 (1.3) 60.8 (1.3) 66.1 (1.3) 72.3 (1.3)	78.1 (1.2) 81.2 (1.1) 90.9 (0.9) 94.4 (0.6)	97.3 (0.4) 97.9 (0.4) 99.4 (0.2) 98.6 (0.3)
low-altitude resident	0.25 0.5 1.0 2.0	97.1 (0.4) 97.9 (0.3) 98.3 (0.3) 97.9 (0.3)	72.6 (1.3) 79.2 (1.3) 85.9 (1.1) 90.7 (1.0)	98.3 (0.4) 99.0 (0.3) 99.2 (0.2) 99.3 (0.3)	59.1 (1.4) 63.1 (1.5) 69.2 (1.4) 75.5 (1.4)	78.3 (1.4) 85.7 (1.2) 91.5 (1.0) 96.2 (0.7)	98.3 (0.4) 99.0 (0.4) 99.4 (0.2) 99.6 (0.3)
RT (ms)							
high-altitude newcomer	0.25 0.5 1.0 2.0	500 (7) 496 (6) 514 (8) 538 (13)	748 (23) 813 (21) 953 (18) 1,069 (23)	531 (9) 567 (9) 592 (10) 645 (21)	733 (24) 804 (25) 1,020 (27) 1,352 (28)	646 (19) 733(20) 877 (19) 1,050 (22)	540 (11) 560 (10) 610 (12) 670 (15)
low-altitude resident	0.25 0.5 1.0 2.0	469 (8) 481 (6) 496 (9) 528 (15)	809 (26) 838 (24) 911 (20) 1,050 (20)	544 (10) 540 (10) 554 (11) 639 (23))	812 (27) 888 (27) 1,040 (29) 1,340 (31)	721 (21) 790 (22) 883 (21) 1,028 (25)	542 (12) 562 (12) 588 (13) 661 (17)

Mean (SD).

Abbreviations: ACC, accuracy; RT, reaction time; ET, exposure time.

The interaction of ratio and ET was significant [*F* (15, 3105) = 50.899, *p* < 0.001, *η* ^*2*^ *p* = 0.20]. Simple effects analysis showed that, in all four ET conditions, there was a significantly higher correct rate in the arrow-consistent condition than in the inconsistent condition (*p* < 0.01). Consistent with this finding, the correct response rate was significantly higher in the 5:0 and 3:0 groups than when the ratio was 1:0 (*p* < 0.01). In the case of inconsistency, the highest correct rate was found in the case of a 4:1 ratio, followed by the correct rate in the case of a 2:1 ratio, whereas the lowest rate was found in the case of a 3:2 ratio (*p* < 0.01).

The interaction between ET and Group was significant [*F* (3, 621) = 10.377, *p* < 0.01, *η* ^*2*^ *p* = 0.14]. Simple effects analysis showed that 1) when ET was 0.25 s, there was no significant difference in the correct rate between the two groups of subjects (*p* > 0.05), while in several other ET conditions, the correct rate was significantly lower in the high-altitude newcomers than in the low-altitude residents (*p* < 0.05). 2) With prolongation of ET, the correctness rate of the two groups of subjects changed significantly: ACC_(0.25s)_<ACC_(0.5s)_<ACC_(1s)_<ACC_(2s)_ (*p* < 0.01).

The interaction between group, ET and ratio was significant [*F* (15, 3105) = 2.134, *p* < 0.05, *η* ^*2*^ *p* = 0.01]. Simple effects analysis showed that 1) when ET was 0.25 s, the correct rate was significantly lower (*p* < 0.05) for high-altitude newcomers than for low-altitude residents at the ratios of 1:0, 2:1, and 3:0. 2) When the ET was 0.5 s, the correctness rate of high-altitude newcomers at ratios of 2:1 and 4:1 was significantly lower than that of low-altitude residents (*p* < 0.01, *p* < 0.01). 3) When the ET was 2 s, the correctness rate was significantly higher in the case of the 4:1 and 5:0 ratios for the low-altitude residents than for the high-altitude newcomers (*p* < 0.01, *p* < 0.05).

A 2 (group) × 4 (ET) × 6 (ratio) repeated-measures ANOVA was performed on the reaction times. The results showed that the main effect of the group was not significant (F < 1). The main effect of ET was significant [*F* (3, 621) = 294.059, <0.001, *η* ^*2*^ *p* = 0.58] and the main effect of Ratio was significant [*F* (5, 1035) = 1041.640, *p* < 0.001, *η* ^*2*^ *p* = 0.83].

The interaction of ratio and group was significant [*F* (5, 1035) = 3.889, *p* < 0.05, *η* ^*2*^ *p* = 0.20]. Simple effects analysis revealed that 1) the reaction times of low-altitude residents were significantly shorter (*p* < 0.05) than those of high-altitude newcomers in the consistent arrow condition, whereas there was no significant difference between the reaction times of the two groups of subjects in the inconsistent arrow condition. 2) Participants in both groups had significantly shorter reaction times for arrow consistency than for inconsistency (*p* < 0.05). Consistent with this, the reaction times were the shortest at a ratio of 1:0 and significantly shorter at a ratio of 3:0 than at 5:0 (*p* < 0.05). In the case of inconsistency, the shortest reaction time was observed at a 4:1 ratio, followed by a 2:1 ratio, whereas the longest reaction time was observed at a 3:2 ratio (*p* < 0.05).

The interaction between ET and group was significant [*F* (3, 621) = 3.632, *p* < 0.05, *η* ^*2*^ *p* = 0.20]. A simple effects analysis revealed that 1) there was no significant difference in the response time between the two groups of subjects in all ET conditions (*p* > 0.05). 2) The comparison of reaction times between the two groups of subjects in the four ET conditions was RT_(0.25s)_<RT_(0.5s)_<RT_(1s)_<RT_(2s_) (*p* < 0.001).

The interaction between ratio and ET was significant [*F* (15, 3105) = 121.754, *p* < .001, *η* ^*2*^ *p* = .37]. A simple effects analysis showed that 1) when the ratio was 1:0, there was no significant difference (*p* > .05) at the time of response, regardless of the ET conditions. 2) When the ratio was not 1:0, the subject’s reaction time varied with ET as follows: RT_(0.25s)_<RT_(0.5s)_<RT_(1s)_<RT_(2s)_ (*p* < 0.01).

The interaction between ratio, ET and group was significant [*F* (15, 3105) = 2.661, *p* < 0.01, *η* ^*2*^ *p* = 0.01]. Simple effects analysis showed that 1) when ET was 0.25 s, response times were significantly shorter in high-altitude newcomers than in low-altitude residents in the Ratio 3:0 case (*p* < 0.05), and significantly longer in the 3:2 and 4:1 ratios (*p* < 0.001, *p* < 0.01). 2) When the ET was 0.5 s, the reaction time was significantly longer in high-altitude newcomers than in low-altitude residents at a ratio of 3:2 (*p* < 0.05).

The results of Study 1 showed that, relative to low-altitude conditions, CCC and task correctness were significantly reduced in newcomers exposed to chronic high-altitude hypoxia; therefore, the hypothesis was validated.

## 4 Study 2. effect of HBO intervention

### 4.1 Methods

#### 4.1.1 Participants

In Study 2, the minimum sample size was 22 based on G*power’s standard of repeated-measures variance. The participants were 56 students, all of whom grew up at low altitudes and entered high altitude (3,650 m) for the first time in their adult lives for 2 years. The participants were randomly divided into HBO and control groups. The HBO group consisted of 14 males and 14 females, aged 18–22 years. The control group consisted of 15 males and 13 females, aged 18–22 years, and there were no significant differences between the two groups of participants in terms of sex and age (*p* > 0.05). All participants had normal or corrected visual acuity and were right-handed, signed an informed consent form before the experiment, and received some remuneration at the end of the experiment.

#### 4.1.2 Hyperbaric oxygen, HBO

Study II used a soft oxygen chamber (NBYY-HYDT-003) for hyperbaric oxygen intervention, which was oval in shape, 2.13 m high and 2.55 m wide, with a lower surface area of 7.88 m^2^. The intervention pressure was 0.11 MPa, oxygen concentration was 25%, and oxygen flow rate was 10 L/min. The ramp-up time of a single oxygen intervention was 40 min, the oxygen was inhaled for 30 min during the constant-pressure phase, and the oxygen was released after 20 min of decompression to be tested out of the chamber. Participants in the HBO group received diffused oxygen in the chamber. The entire process lasted approximately 90 min each time. Participants in the HBO group were required to complete 15 times 90-min HBO interventions over a month, with one intervention every other day. The control group participants were placed in the same enclosed chamber without receiving HBO intervention, unbeknownst to them. SpO_2_ was measured before and after the hyperbaric oxygen intervention in all participants.

#### 4.1.3 Procedure

For all participants in Study 2, there were no differences in the factors influencing sex, age, or visual acuity. All subjects completed one MFT-M task prior to the hyperbaric intervention, and the experimental group completed 15 hyperbaric interventions over the course of 1 month. At each intervention, the partial pressure of oxygen peaked after 40 min of boosting, after which oxygen was administered at a constant pressure for 30 min and finally decompressed for 20 min out of the chamber. Participants in the HBO group completed an MFT-M task within 24 h of exiting from the hyperbaric intervention in the 15th session, and the control group did not perform any intervention, with the same time interval between the two tests for participants in both groups. Study 2 used a four-factor mixed experimental design as follows: 2 (group) × 4 (ET) × 6 (ratio) × 2 (time) 1). Group: divided into two conditions of hyperbaric oxygen intervention and no intervention, that is, HBO group and control group 2). Ratio: 1:0, 2:1, 3:1, 3:2, 4:1, 5:0 3). ET: 0.25, 0.5, 1, 2 s 4) Time: before and after hyperbaric intervention, for the experimental group divided into 15 hyperbaric interventions before and 15 hyperbaric interventions after. Group was the between-group variable, and the remaining factors were all within-group variables.

### 4.2 Results

**FIGURE 4:**
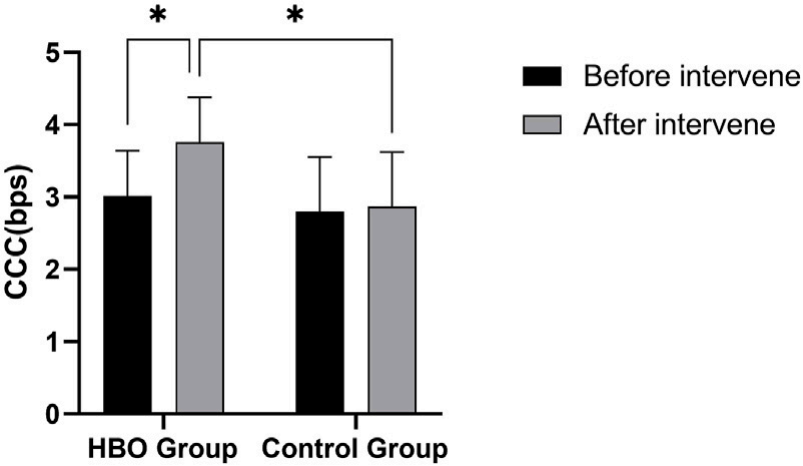
CCC size of the HBO and control groups before and after the intervention. Note: *p < 0.05.

The results of a 2 (group) × 2 (time) repeated-measures ANOVA on CCC (Figure 4) showed that the group main effect was significant [*F* (1, 54) = 7.444, *p* = 0.009, *η* ^*2*^ *p* = 0.121], and the time main effect was significant [*F* (1, 54) = 16.224, *p* < 0.000, *η* ^*2*^ *p* = 0.231]. The CCC size was 2.80 (SD = 0.75) bps before and 2.87 (SD = 0.75) bps after the intervention in the control group and 3.02 (SD = 0.62) bps before and 3.57 (SD = 0.62) bps after the intervention in the HBO group.

Time*group interaction was significant [*F* (1, 54) = 9.557, *p* = 0.003, *η* ^*2*^ *p* = 0.150]. Simple effects analysis showed no significant difference in CCC between the HBO and control groups before the intervention (*p* = 0.229). CCC was significantly higher in the HBO group than in the control group after 15 hyperbaric interventions (*p* < 0.000), and CCC was significantly higher in the HBO group after 15 hyperbaric interventions than before the intervention (*p* < 0.000), whereas no significant difference was produced in the control group (*p* = 0.511). The interaction results showed that the HBO and control groups were reasonably grouped and that the hyperbaric intervention significantly improved the CCC in the HBO group.

The results of the repeated measures ANOVA of 2 (group) × 6 (ratio) × 4 (ET) × 2 (time) for ACC showed significant Group main effects [*F* (1, 54) = 4.367, *p* = 0.041, *η* ^*2*^ *p* = 0.075], significant time main effects [*F* (1,54) = 8.616, *p* = 0.005, *η* ^*2*^ *p* = 0.138], ratio main effect was significant [*F* (1, 54) = 13.917, *p* < 0.000, *η* ^*2*^ *p* = 0.938], ET main effect was significant [*F* (1, 54) = 13.917,*p* < 0.000,*η* ^*2*^ *p* = 0.698].

The time*group interaction was significant [*F* (1, 54) = 4.416, *p* = 0.040, *η* ^*2*^ *p* = 0.076], and simple effects analysis showed that ACC was not significantly different between the HBO and control groups before the intervention (*p* = 0.312). The HBO group had a significantly higher ACC than the control group after 15 hyperbaric interventions (*p* = 0.008), and the HBO group had a significantly higher ACC after 15 hyperbaric interventions than before the intervention (*p* = 0.001), whereas the control group did not produce a significant difference (*p* = 0.558). The interaction results showed that the HBO and control groups were reasonably grouped, and that the hyperbaric intervention significantly increased the accuracy rate of the HBO group.

The interaction of time*ET was significant [*F* (1, 54) = 3.075, *p* = 0.032, *η* ^*2*^ *p* = 0.054], simple effects analysis revealed that 1) the pre-intervention correctness rate was significantly lower than the post-intervention rate at ET of 0.25 s versus 0.5 s (*p* = 0.007, *p* = 0.012). 2) When the ET was 1 s versus 2 s, there was no significant difference in the correctness of the task before and after the intervention.

The interaction between time*ratio*group was significant [*F* (1, 54) = 3.683, *p* = 0.013, *η* ^*2*^ *p* = 0.064]. Simple effects analysis showed that 1) there was no significant difference in correctness between the two groups in the different Ratio conditions before the intervention. 2) The HBO group had significantly higher correct rates than the control group when the ratios were 3:0 and 4:1 after the intervention (*p* = 0.001, *p* = 0.044). The results of the interaction showed that the hyperbaric intervention significantly increased task accuracy in the HBO group.

A repeated-measures ANOVA of 2 (group) × 6 (ratio) × 4 (ET) × 2 (time) at the reaction time showed that the group main effect was not significant [*F* (1, 54) = 0.364, *p* = 0.557, *η* ^*2*^ *p* = 0.006], and no significant difference was seen between the HBO and control groups. The main effect of time was significant [*F* (1, 54) = 10.253, *p* = 0.002, *η* ^*2*^ *p* = 0.160], the main effect of ratio was significant [*F* (1, 54) = 297.530, *p* < 0.000, *η* ^*2*^ *p* = 0.846], and the main effect of ET was significant [*F* (1, 54) = 179.160, *p* < 0.000, *η* ^*2*^ *p* = 0.768].

The time*ratio interaction was significant [*F* (1, 54) = 3.171, *p* = 0.035, η2*p* = 0.055]. Simple effects analysis showed that 1) pre-intervention response times were significantly higher than post-intervention response times in all ratio conditions (*p* < 0.05). 2) There was a significant difference (*p* < 0.05) in response time between the two groups before and after the intervention and between the different ratio conditions. The ratio × ET interaction was significant [*F* (1, 54) = 78.875, *p* < 0.000, η2*p* = 0.594], and no other significant effects were observed.

The results of Study 2 showed that the CCC in the HBO group was significantly higher after 15 hyperbaric interventions than before (*p* = 0.005), while there was no significant difference in the control group (*p* = 0.972). The HBO group had significantly higher correct task rates than the control group after the intervention (*p* = 0.001).

## 5 Discussion

The current study measured the CCC of various subjects with the help of the MFT-M task, and the resulting data were quantified and analyzed. We explored the reduction in CCC in newcomers by prolonged exposure to high-altitude hypoxia and the effect of hyperbaric interventions on overall cognitive control processes by affecting CCC.

Study 1 showed that CCC was significantly higher in the low-altitude residents than in high-altitude newcomers, suggesting that long-term exposure to high-altitude hypoxia can impair CCC in newcomers. In addition to structural changes due to chronic hypoxia, the hypoxic environment of the plateau may lead to a prolonged state of stress in the organism with high executive control requirements and excessive occupation of attentional resources, causing the human brain to process less information per unit time (Wang et al., 2014). Related studies have shown that long-term exposure to high-altitude hypoxic stress leads to structural changes in brain function during transplantation, resulting in reduced cognitive control in newcomers (Wu et al., 2016).

Further analysis of the performance of the two groups of subjects in the MFT-M task showed that when the ET was 0.5, 1, and 2 s, the low-altitude residents had a higher correct rate than the high-altitude newcomers. These results were consistent with the study by Ma et al. (2019). This can be explained by the effect of long-term high-altitude exposure on working memory, where the lack of attentional resources of high-altitude newcomers during task completion leads to the impairment of information maintenance, which affects the correct response rate. The correct rates of high-altitude newcomers under several ratios at the random level were significantly lower than those of low-altitude residents, suggesting that there is indeed an inhibitory effect of a low-oxygen environment on the visual recognition sensitivity of migrants.

In the case of consistent arrows, the reaction time of the low-altitude residents was significantly shorter than that of the high-altitude newcomers, probably due to the long-term exposure of the high-altitude newcomers to hypoxia, which lowers the visual sensitivity and slows down the cognitive speed, and the hypoxia slows down the speed of brain thinking and makes it take longer to judge the stimuli, resulting in a longer reaction time (Nakata et al., 2017). In contrast, when the arrows did not match and there was no significant difference between the two groups, it is possible that the floor effect made the difference between the two groups of subjects insignificant as task difficulty increased.

The results of Study 2 showed that 15 hyperbaric interventions significantly increased the correct rate and CCC in the HBO group compared with the control group, and the correct rate and CCC in the HBO group were significantly greater after the intervention than before the intervention. The CCC in the HBO group after the intervention (3.57, SD = 0.62) bps was slightly greater than that in the low-altitude resident group in Study 1 (3.35, SD = 0.72) bps, suggesting that HBO can effectively improve cognitive ability and that CCC impairment caused by high altitude hypoxia can be improved by oxygenation, and that the cognitive ability of high-altitude newcomers may recover to some extent after returning to lower altitudes.

Cognitive enhancement studies of HBO interventions in healthy young adults have shown that HBO significantly enhances memory function, especially spatial working memory. HBO interventions have also been found to significantly enhance connectivity in the limbic system of subcortical areas (Yu et al., 2015), which mainly includes the thalamus, cingulate gyrus, and hippocampus, and is associated with emotion, memory, learning, and visuospatial skills (Gitelman et al., 1999) that are responsible for higher cognitive functions. In the present study, CCC was significantly improved in the HBO group after 15 HBO interventions, reflecting, to some extent, the holistic function of the brain regarding cognitive control. The increased cognitive control capacity after oxygenation indicated improved cognitive control and more complete and close integration connections between brain regions. After HBO intervention, the oxygen level in the blood increases rapidly and is delivered mainly to hypoxic areas of vital organs, preventing impaired brain tissue function, enabling the preferential selection of important information for response judgments and increased processing resources within a restricted timeframe, thus enhancing cognitive function. However, we do not believe that the improvement effect of the 15 HBOs sessions will last because short-term HBOs sessions are not sufficient to change the improvement structure. However, hyperbaric oxygen therapy (HBOT) may have some potential side effects. McMonnies (2015) discussed the possibility of ocular complications or contraindications associated with HBOT due to increased tissue concentrations of reactive oxygen species. Heyboer et al. (2017) conducted a retrospective chart review on the effect of HBOT on blood pressure in patients undergoing treatment, demonstrating that absolute rises in blood pressure can occur as a result of HBOT therapy. Overall, the literature suggests that HBOT has diverse applications and potential benefits in various medical conditions, but it is essential to consider potential side effects and contraindications associated with this therapy.

Further analysis of the performance of the two groups of participants in the MFT-M task, in which the number and consistency of arrows for each trial condition were known, showed a decrease in accuracy with increasing information entropy when the upper limit of cognitive control processing was reached or exceeded. The results indicated that the accuracy of the control group was lower than that of the HBO group under the same experimental conditions. However, the information entropy of the task was greater at an arrow ratio of 3:2, which caused the greatest conflict for the participants, such that the accuracy of the two groups at presentation times of 250 ms and 500 ms was close to a 50% random probability. Correctness and reaction time were strongly influenced by presentation time and task difficulty, but were not the main indicators in this study, but it could still be judged that when the task was more difficult (short presentation time and close number of left and right arrows), the correctness rate was significantly higher in the post-intervention subjects than in the pre-intervention as well as in the control group. Under any condition, a presentation time of 2 s was the most easily judged difficulty condition, and the correct rate almost appeared as a ceiling effect, whereas the correct rate of the rest of the conditions was in the middle.

In conjunction with our previous studies showing that chronic high-altitude exposure affects a variety of cognitive control functions, high-altitude newcomers have reduced attentional resources due to persistent hypoxia, which puts the body in a state of chronic stress, including alerting, orienting, orienting, and executive control, which affects other brain networks that calculate priorities when acquiring awareness or output (Fan et al., 2005; Xuan et al., 2016). When faced with information, the brain needs to make effective selections, allocate resources, and prioritize the amount of information. The total amount of human cognitive resources is limited under high-altitude hypoxia, and when the amount of uncertain information processed consumes resources that do not exceed the total amount, the task can be performed accurately; when the amount of uncertain information processed exceeds, it triggers guessing leading to random responses. The effective execution of uncertain information and the allocation of cognitive resources is reduced, and more cognitive resources are put on guessing, thus reducing the correct rate of the task and response time, further reducing CCC.

The 15-session HBO intervention increased the amount of uncertain information processing and total amount of cognitive resources. When faced with uncertain information, the brain allocates more cognitive resources to the judgment of uncertain information rather than to guessing, thus increasing the effectiveness and accuracy of task execution, as evidenced by an increase in the ACC and CCC, and improving the overall cognitive level of the brain. We hypothesize that for the amount of uncertain information, the presentation time of uncertain information is more likely to be improved by HBO, which may reveal that cognitive resources are first allocated to the grasp of overall presentation time rather than directly to the search of uncertain information conflict situations, and thus subsequent studies can further explore the neural mechanisms underlying the functional state of the human brain and the functional connectivity between brain regions after HBO intervention.

## 6 Limitations

Some pressing issues still need to be addressed in the current study. The main limitations are as follows:

First, cognitive control is considered a central channel for information transfer between different sensory channels and cognitive domains, and the present experiment estimated CCC using only visuospatial processing behavioral tasks in the cognitive decision-making domain. In future, we plan to estimate CCC using other cognitive pathways.

Second, Study 2 focused on the effects of multiple HBO interventions on cognitive control, but did not analyze the prognostic effects, how long the improvement in CCC brought about by HBO could last, and what changes in CCC levels would occur when returning to high-altitude conditions after the intervention are not yet clear and need further confirmation. The participants were all high-altitude immigrants for 2 years, regardless of whether the exposure time was sufficient. The CCC in the HBO group returned to the plains level after hyperbaric intervention, indicating that the cognitive impairment caused by high-altitude exposure will revert on its own after the immigrants return to lower altitudes, and the follow-up study will focus on the longer-term reversion effect and the neural remodeling mechanism.

## 7 Conclusion

Long-term exposure to the hypoxic and low-pressure environment of the high-altitude does produce a certain degree of impairment in the CCC of the newcomers, and the 15 hyperbaric oxygen interventions improved the CCC of the individuals, thus improving the cognitive control function of high-altitude newcomers. Hyperbaric oxygen intervention is an effective way to improve the cognitive function of high-altitude residents, which is conducive to preventing and improving the cognitive impairment of high-altitude residents, improving their quality of life and work efficiency, and improving the development of high-altitude areas.

## Data Availability

The raw data supporting the conclusions of this article will be made available by the authors, without undue reservation.
